# Spatial and Temporal Occurrence of Blue Whales off the U.S. West Coast, with Implications for Management

**DOI:** 10.1371/journal.pone.0102959

**Published:** 2014-07-23

**Authors:** Ladd M. Irvine, Bruce R. Mate, Martha H. Winsor, Daniel M. Palacios, Steven J. Bograd, Daniel P. Costa, Helen Bailey

**Affiliations:** 1 Marine Mammal Institute, Department of Fisheries and Wildlife, and Coastal Oregon Marine Experiment Station, Oregon State University, Hatfield Marine Science Center, Newport, Oregon, United States of America; 2 Cooperative Institute for Marine Ecosystems and Climate, Institute of Marine Sciences, Division of Physical and Biological Sciences, University of California Santa Cruz, Santa Cruz, California, United States of America; 3 NOAA/NMFS/SWFSC/Environmental Research Division, Pacific Grove, California, United States of America; 4 Ecology and Evolutionary Biology, Long Marine Laboratory, University of California Santa Cruz, Santa Cruz, California, United States of America; 5 Chesapeake Biological Laboratory, University of Maryland Center for Environmental Science, Solomons, Maryland, United States of America; Texas A&M University-Corpus Christi, United States of America

## Abstract

Mortality and injuries caused by ship strikes in U.S. waters are a cause of concern for the endangered population of blue whales (*Balaenoptera musculus*) occupying the eastern North Pacific. We sought to determine which areas along the U.S. West Coast are most important to blue whales and whether those areas change inter-annually. Argos-monitored satellite tags were attached to 171 blue whales off California during summer/early fall from 1993 to 2008. We analyzed portions of the tracks that occurred within U.S. Exclusive Economic Zone waters and defined the ‘home range’ (HR) and ‘core areas’ (CAU) as the 90% and 50% fixed kernel density distributions, respectively, for each whale. We used the number of overlapping individual HRs and CAUs to identify areas of highest use. Individual HR and CAU sizes varied dramatically, but without significant inter-annual variation despite covering years with El Niño and La Niña conditions. Observed within-year differences in HR size may represent different foraging strategies for individuals. The main areas of HR and CAU overlap among whales were near highly productive, strong upwelling centers that were crossed by commercial shipping lanes. Tagged whales generally departed U.S. Exclusive Economic Zone waters from mid-October to mid-November, with high variability among individuals. One 504-d track allowed HR and CAU comparisons for the same individual across two years, showing similar seasonal timing, and strong site fidelity. Our analysis showed how satellite-tagged blue whales seasonally used waters off the U.S. West Coast, including high-risk areas. We suggest possible modifications to existing shipping lanes to reduce the likelihood of collisions with vessels.

## Introduction

The blue whale (*Balaenoptera musculus*) population in the eastern North Pacific (ENP) was depleted by commercial whaling in a manner similar to populations that were overexploited in other parts of the world [1]. While there is some evidence that the population was increasing in the late 1900s [2], recent population estimates for waters off California, Oregon, and Washington based on line-transect methods have ranged from about 500 to nearly 2000 animals [3], suggesting that the proportion of the population foraging off the U.S. West Coast varies inter-annually. Current mark-recapture estimates based on photo-identification studies indicate that the population size is about 2500 whales [4] and that its distribution may have expanded north into waters off British Columbia and the Gulf of Alaska in recent years [5]. It was expected that the ENP blue whale population would begin to recover following the protections established by the International Whaling Commission in 1966 [6], so the lack of evidence of substantial population growth during the past decades may indicate their recovery is being impeded, possibly by human impacts, either indirectly through food chain interactions [7], or directly from physical interactions such as noise [8], [9] or ship strikes [10]. It is therefore essential that areas of importance to ENP blue whales off the U.S. West Coast are identified so that appropriate management actions may be taken for this endangered species.

Most current estimates of blue whale distribution and population density in the ENP are based on photo-identification and line-transect data from shipboard surveys [2], [3]. However, tracking whales with satellite tags has the advantage of higher temporal resolution than ship-based surveys, allowing for a more detailed understanding of how the distribution of tagged animals changes over time, while also providing better spatial resolution than can be achieved by other techniques like passive acoustic monitoring [11], . Tracking data from individual whales allow the location and extent of important areas to be identified based on frequency of use, without the limitations of pre-determined survey areas or times. Here we present results from a large, multi-year set of blue whale satellite tracking data, collected off the U.S. West Coast, to determine which areas were most important to tagged whales and whether those areas changed inter-annually during the study period. These results provide valuable information on the home range and core areas of blue whales on their feeding grounds off the U.S. West Coast, which will help better inform management agencies.

## Methods

### Ethics Statement

This research was conducted under U.S. National Marine Fisheries Service (NMFS) permit numbers 841 (years 1993–1998), 369-1440 (years 1999–2004), and 369-1757 (years 2005–2008), authorizing the close approach and deployment of implantable satellite tags on large whales which are protected by the 1972 Marine Mammal Protection Act, and, in many cases, the 1973 Endangered Species Act. All tagging procedures described in the listed permits, and used in this manuscript, were subjected to an internal NMFS and external review by veterinarians and other marine mammal researchers prior to approval. In addition, this study was carried out in strict accordance with the recommendations of the Oregon State University Institutional Animal Care and Use Committee, composed of veterinarians and other university administrators.

The impacts of tagging on cetaceans relative to the conservation value of the information that this technique provides has been reviewed in several fora, beginning with a multi-agency (U.S. Marine Mammal Commission, NMFS, and U.S. Office of Naval Research) workshop in 1987 [14] and continuing through a 2011 report by the Scientific Committee of the International Whaling Commission [15]. These committees and workshops have repeatedly determined that, for highly endangered species, the level of health risk from implantable satellite tags is sufficiently low compared to the high potential conservation value of the data, and as such has encouraged the conduct of tagging studies. While we agree with these assessments, we do not discount that some amount of pain or discomfort may be associated with the implantation of satellite tags in large cetaceans. However, there is a lack of understanding of the level of pain felt by a whale swimming with an implanted tag for extended periods of time [16] or whether this impedes its natural behaviors in a significant way [17]. Research on effects of implantable tags has generally been limited to short-term behavioral responses [17], or long-term reproductive rate [18]. Nevertheless, several whale species have been tracked with this technology to known (as well as previously unknown) areas of concentration [19], [20], and using recognized migration corridors [21], including historical areas of sightings and whaling catch records [22], [23], indicating that the long-term movements of tagged whales are consistent with other, non-tagged whales.

### Satellite tracking

Satellite monitored radio tags were attached to 171 blue whales in the ENP during 15 years ranging from 1993 to 2008. While the specific components and construction of the tags varied over the years, the tags generally consisted of a Telonics UHF transmitter with batteries housed in a stainless steel cylinder that was attached to the whale using either two sub-dermal attachments (surface-mounted style) or one four-bladed attachment on the end of the housing (implantable style). Tags were also equipped with a salt-water conductivity switch to prevent them from transmitting while underwater. Details about the types and evolution of tag designs, including the exact dimensions of each type and the deployment methods can be found in Mate et al. [24].

Tags were mainly deployed along the California coast, either at the western part of the Santa Barbara Channel (n = 113) or in the Gulf of the Farallones (n = 41), with a few deployed near Cape Mendocino (1998, n = 2), Big Sur (2005, n = 5) and near Monterey Bay (2005, n = 10). Tags were attached 1–3 m forward of the whales’ dorsal fin, near the midline, from a small (<7 m) rigid-hulled inflatable boat, using either a 68-kg modified Barnett crossbow (1993–1995, 1998–2002), or the Air Rocket Transmitter System (ARTS, 2004–2008), a modified line-throwing gun using compressed air [25]. Whales were visually inspected for evidence of previous tag attachment during an approach to identify if the same individual was tagged in multiple years.

For this study, whales were located through visual searches in nearshore areas of known aggregation rather than through structured surveys. Once located, whales were approached and tagged opportunistically, and therefore particular individuals were not specifically selected for tagging. For these reasons, there is a possibility, though we believe it is unlikely, that individual variability of behaviors like surfacing rate or duration at the surface might make some whales more likely to be tagged. Additionally, the overall spatial distribution of the tagged whales presented here may not represent the entire ENP blue whale population.

Tags were programmed with one of three duty cycles: transmitting every day; transmitting every other day; or transmitting every day for the first 90 days, then transmitting every other day for the remainder of the tag life. On scheduled transmission days, the tags were programmed to transmit every 10 s (when ‘dry’, i.e. at the surface) during four 1-h periods with the transmission periods scheduled to coincide with the most likely times a satellite was overhead. Locations were calculated by Service Argos from the Doppler shift of the transmissions when three or more messages reached a satellite during a single pass overhead. Each location was assigned an estimated accuracy classification (in descending order of accuracy: 3, 2, 1, 0, A, B, Z) based on the timing and number of transmissions received during a satellite pass [26].

A Bayesian switching state-space model [27] was applied to the raw, unfiltered locations from each track to account for satellite location errors based on the Argos quality classes and to provide regularized tracks with one estimated location per day [27], [28]. This also provided an estimated behavioral state for each location as either transiting, when the whale moves in a relatively rapid, linear path, or Area Restricted Search (ARS), when locations are clustered. Since this study focused on the summer foraging grounds off the U.S. West Coast, ARS locations likely correspond to foraging behavior. A full description of this model and the tracks from tags deployed in 1993 to 2007 (n = 159) are presented in Bailey et al. [28]. Here we additionally present new tracking data from tags deployed in 2008 (n = 12), and analyze the tracks on a finer scale focusing on the whales’ movements on the foraging grounds off the U.S. West Coast.

### Data Analysis

The U.S. Exclusive Economic Zone (EEZ) consists of ocean waters extending out to 200 nautical miles from the coastline [29]. The portions of tracks that occurred inside the EEZ were extracted and used in all further analyses. The Julian day when a whale departed the EEZ (number of days from 1 January) was recorded to help identify the seasonality of blue whale occurrence in U.S. waters.

Home ranges created from tracking data have been used in a variety of wildlife studies to describe the geographically restricted area used by animals and how it relates to the distribution and abundance of a population [30], habitat selection [31], [32], and predator-prey dynamics [33] among others topics [34]. The modern statistical modelling of home ranges implemented in this study uses the location data to estimate a probability density function that describes the likelihood of an animal being present at a given point within the home range [34], [35], [36]. For each track, kernel home ranges were created from EEZ locations using the least-squares cross-validation (LSCV) bandwidth selection method [37], [38]. The kernel analysis was implemented using the adehabitat package [39] in R (v 2.11.1) [40]. The 90% (home range, HR) and 50% (core area of use, CAU) isopleths were produced for each track with 30 or more estimated locations [41] and all portions that overlapped land were removed. The 90% contour was chosen for the HR because the 95% contour can include more extraneous or transitory locations [42].

HR and CAU areas were calculated for each individual and tested for dependence on track duration using linear regression. The variation in HR and CAU areas among years was then tested using a linear regression to identify potential inter-annual variability. The HRs and CAUs were then compared among whales and the number of individuals sharing overlapping areas was calculated for all tracks and for tracks within each year. We used the number of overlapping individual HRs and CAUs in an area as a metric to characterize how much it was used by the tagged whales. Locations of designated shipping lanes were overlaid on the HR and CAU maps to identify possible areas of increased vessel collision risk.

To confirm the areas of highest use shown by the HR and CAU areas were not a product of tagging location bias, we created a gridded utilization distribution using all the locations that accounted for variation in track duration, and reduced the bias of the tagging location by implementing a weighting scheme developed in Block et al. [43]. This scheme weighted each location by the inverse of the number of individuals that had locations on the same relative day up to the 85th percentile of track lengths, beyond which location weights were set equal to the weight at that threshold. These location weights were summed within each quarter-degree latitude/longitude grid cell to provide a relative estimate of habitat use.

The timing of blue whale presence in U.S. waters is important information for managers trying to estimate the likelihood of human-whale interactions, so a histogram of the total number of locations by week was used to characterize the seasonality of blue whale occurrence in the EEZ. The latitudes of all locations were also grouped and plotted by week to better visualize how the north-south distribution of the tagged whales varied over time. Departure from the EEZ may be initiated based on feeding success during the summer, so we tested for differences in EEZ departure day by year using an ANOVA, and for the effect of foraging behavior on the EEZ departure day using a linear regression with a range of metrics based on the ARS locations classified by the state-space model. We calculated the number of ARS patches (>3 consecutive ARS locations) per track, average ARS patch duration (average number of consecutive ARS locations), longest ARS patch duration, duration of the last ARS patch prior to departure, and overall fraction of all track locations that were classified as ARS behavior, to test for an effect on the departure day.

## Results

Tag duration increased as tag attachment types and deployment methods changed. Surface-mounted tags (1993–1995) were the shortest-lived tags with a median duration of 0.5 d (range: 0–81 d, n = 52). Duration dramatically improved for implantable tags deployed by crossbow (median = 58 d, range: 0–306, n = 44), and ARTS-deployed implantable tags were the longest lasting (median = 85 d, range: 0–504 d, n = 75, Table 1). Fifty-three individual tracks from eight years had 30 or more locations after applying the state-space model and extracting locations inside the EEZ (Table 1). A few tracks required special treatment because the animals left and re-entered the EEZ. One tag (No. 3300840 from 2004) lasted 504 d, so the initial movements within the EEZ were recorded as a 2004 track, while its movements in the EEZ the following spring-fall were recorded as a 2005 track. Five tracks left the EEZ and returned later after having traveled north to Vancouver Island, Canada, and Alaska, and in one case south to the southern tip of Baja California, Mexico (Figures S1 & S2). The resulting gaps in the tracks within the EEZ could bias the home-range estimates, so only the longest continuous series of locations within the EEZ was used from these tracks. This criterion excluded <5% of the locations for all but two of the tracks and the locations of core areas were unchanged for all tracks.

**Table 1:** Summary of satellite tagged blue whale tracks.

Year	Number of Tags w/> = 30 US EEZ locs	Number of Tags w/Departure Day	
1993	0	0	Surface Mount Crossbow Deployed
1995	0	3	median duration = 0.5 d, range: 0–81 d, n = 52
1998	3	4	Implantable Crossbow Deployed
1999	7	11	median duration = 58 d, range: 0–306, n = 44
2000	1	2	
2004	11	5	
2005	12	6	Implantable ARTS Deployed
2006	2	4	median duration = 85 d, range: 0–504 d, n = 75
2007	7	8	
2008	10	5	
total	53	48	

Number of tracks with > = 30 locations in the U.S. Exclusive Economic Zone waters and the number of tracks that departed U.S. Exclusive Economic Zone waters listed by year. Deployment method and duration summary for all tags deployed is listed to the right.

**Figure 1:**
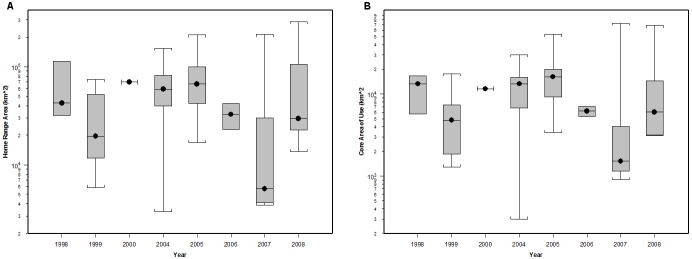
Blue whale 90% Home Range area (A) and 50% Core Area of Use (B). Kernel derived Home Ranges and Core Areas of Use were created from blue whale satellite tracks with > = 30 daily locations inside the U.S. Exclusive Economic Zone. Tags were deployed off California from 1998–2008. Data are presented by year on a log scale and the circles inside the boxes are median values.

**Figure 2:**
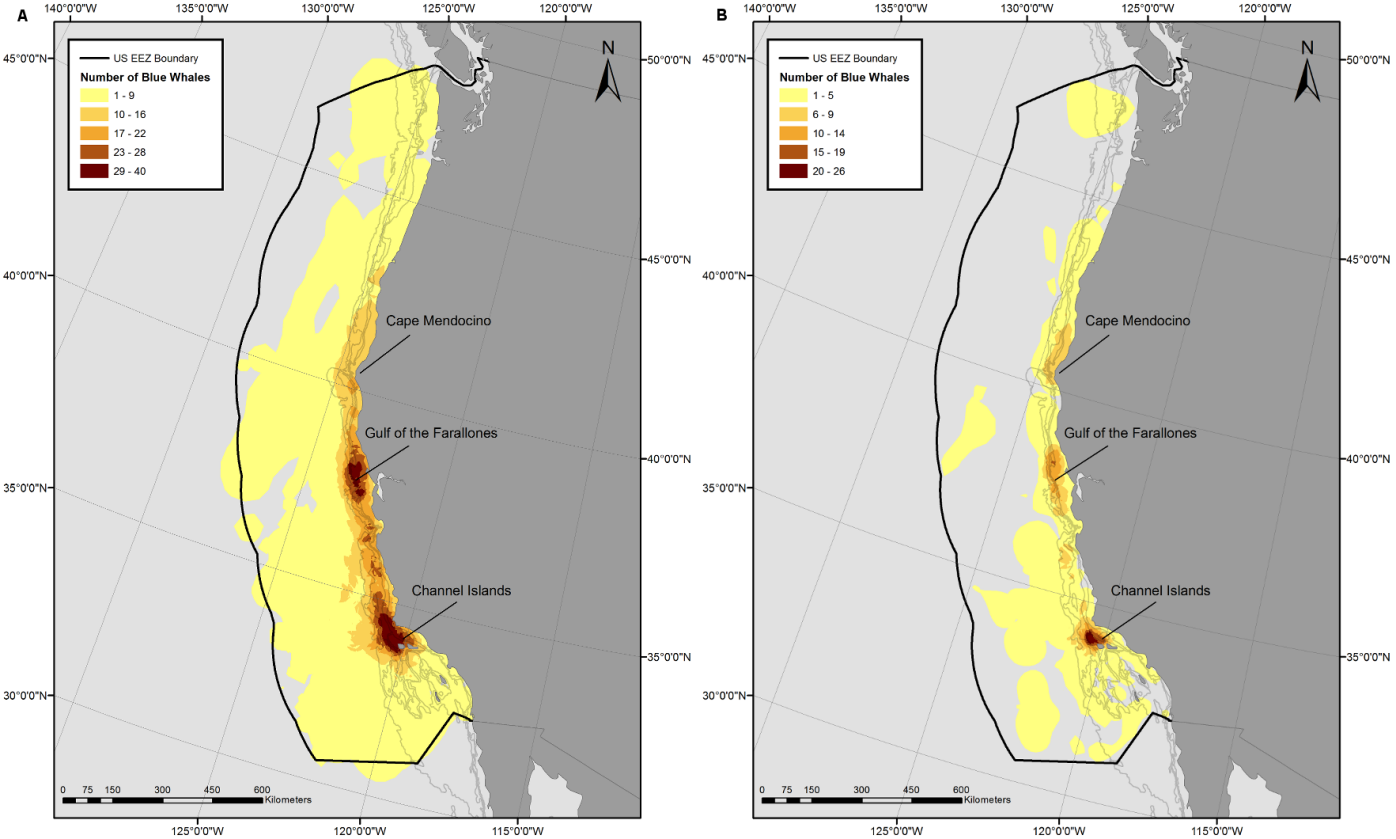
Individual overlapping 90% Home Range areas (A) and 50% Core Areas of Use (B). Kernel derived Home Ranges and Core Areas of Use were created from blue whale satellite tracks with > = 30 daily locations inside the U.S. Exclusive Economic Zone. Shading in the figure represents the number of individual home range and core areas of use that are overlapping in that area. Number of overlapping areas was used as a metric to characterize how much an area was used by the tagged whales. Tags were deployed off California from 1998–2008.

**Figure 3:**
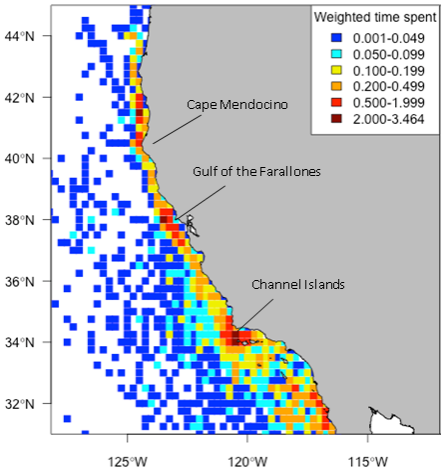
Relative time spent by satellite tagged blue whales in quarter degree grid cells. Relative time spent was calculated from satellite-tagged blue whale tracks using weighted locations to account for unequal tracking durations among individuals. Tags were deployed off California from 1998–2008.

HR and CAU sizes varied considerably between individuals and years (Figure 1), but were not dependent on track duration (p = 0.20 and p = 0.68, respectively, from a log-transformed linear regression). No significant difference was observed among years in HR or CAU size (p = 0.10 and 0.18, respectively, from ANOVA). The combined HRs of all tagged whales covered most of the EEZ (Figure 2A). Areas of highest overlap among individuals were typically near the continental slope between the Channel Islands and the Gulf of the Farallones, California, and in some years extended up to Cape Blanco, Oregon (Figures S3, S4, S5, S6, S7, S8 & S9). CAUs showed a similar distribution of high overlap areas among individuals, but the degree of overlap was much lower (Figure 2B) both in number (as many as 40 HRs overlapping compared to 26 CAUs) and area (33,500 km^2^ area of > = 20 HRs overlapping compared to 846 km^2^ area of > = 20 CAUs overlapping). The area of highest CAU overlap was at the western part of the Channel Islands, with other high overlap areas located near the Gulf of the Farallones and at the northern part of Cape Mendocino. The gridded utilization distribution showed the same pattern with high use on the continental slope and hot spots off California at the Channel Islands, Gulf of the Farallones, and Cape Mendocino (Figure 3), confirming that variable track duration did not bias the pattern of occupancy.

**Figure 4:**
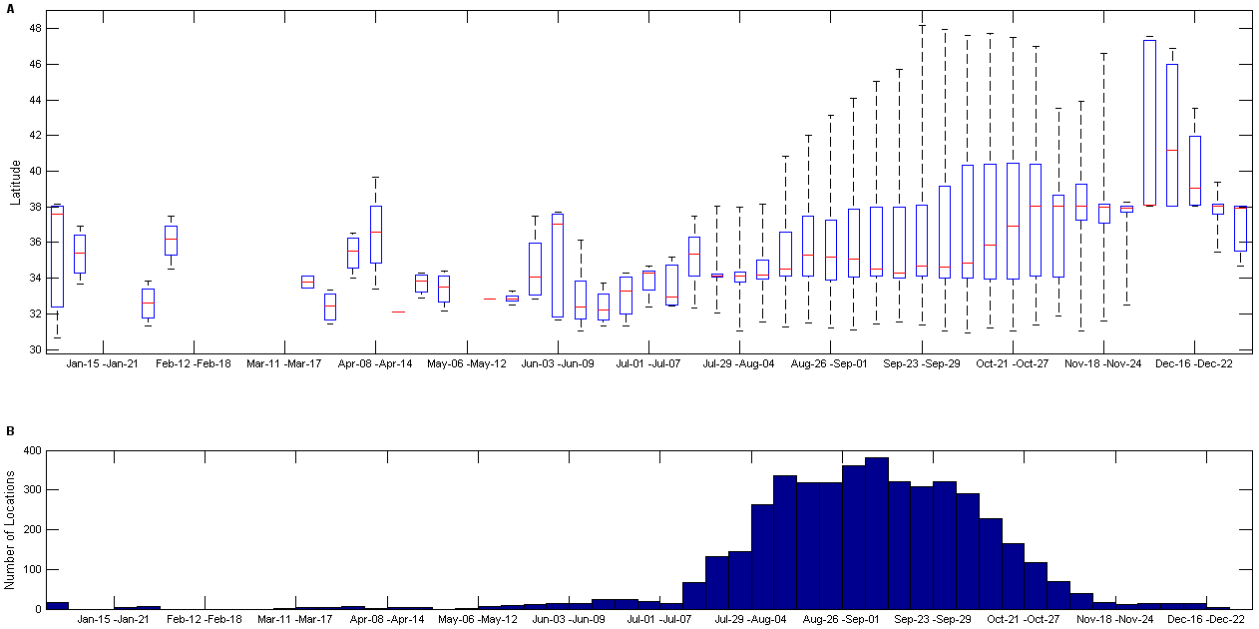
Latitude (Top) and number (Bottom) of blue whale satellite locations plotted by week. Locations used in the figure were from portions of blue whale satellite tracks that occurred within the U.S. Exclusive Economic Zone waters. The red line indicates the median value.

**Figure 5:**
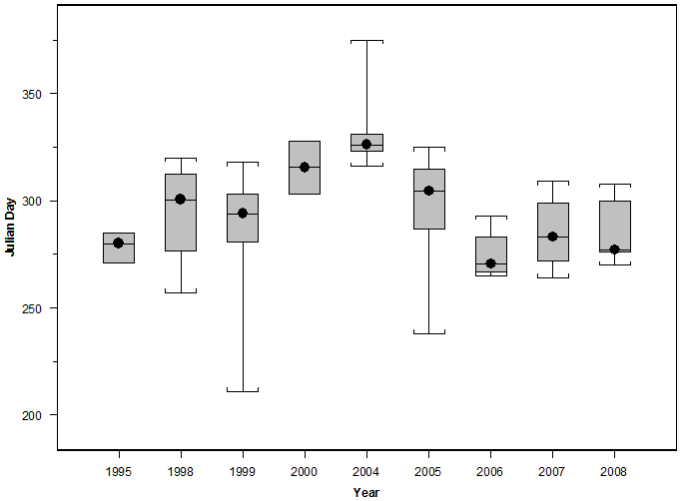
Julian day of U.S. Exclusive Economic Zone departure by satellite tagged blue whales. Circles in the boxes are median values.

While the whale tracks were distributed over a wide area, they tended to occupy the more northerly portion of the range during the latter part of the feeding season in late October-November (Figure 4). The trend was most pronounced in 2005, with 1999 and 2008 showing relatively little northward movement (Figures S10 & S11). Track durations were sufficiently long that EEZ departure days were recorded for 48 tracks in nine years (Table 1). Tracks with recorded departure days did not always meet the 30-d home-range criterion, as some whales began their southerly migration shortly after being tagged. Tagged whales typically departed the EEZ in October (mean = 21 October) although it ranged from as early as late July (in 1999) to as late as 11 January (in 2004, represented as Julian day 375; Figure 5). Departure from the EEZ was significantly later in 2004 than all other years (p = 0.004, F_1,47_ = 8.915, ANOVA), with no significant difference in departure date for the other years (p = 0.775, F_7,40_ = 0.571, ANOVA). No measures of ARS foraging behavior (number of ARS patches/track, average ARS patch duration, longest ARS patch duration, duration of the last ARS patch, and overall fraction of all track locations that were classified as ARS behavior) had a significant effect on the departure date (p = 0.159, 0.668, 0.271, 0.925, and 0.608 respectively from linear regression).

**Figure 6:**
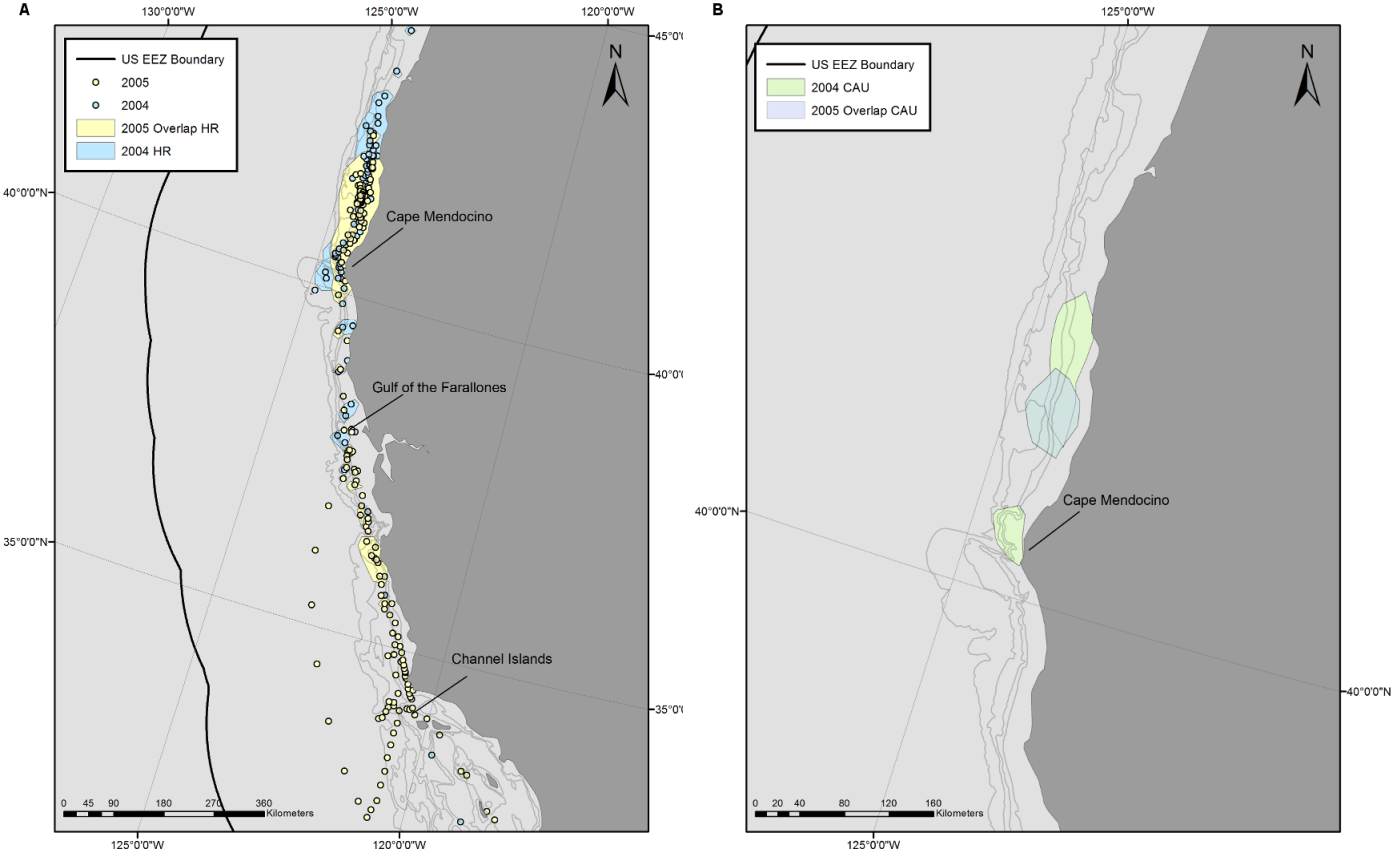
One whale was tracked for 504-year comparisons of space use. Locations within the U.S. Exclusive Economic Zone were recorded from 20 August to 12 November in both 2004 and 2005 for one whale. 90% Home Range (A) and 50% Core Areas of Use (B) were created from the locations to characterize the areas used by the whale in 2004 (blue) and 2005 (transparent yellow to better see overlap). The full summer tracks from 2004 and 2005 are included in the first image for reference.

The multi-year track of whale No. 3300840 allowed us to characterize its entire EEZ range in 2005, as well as any variability in distribution between 2004 and 2005. The whale spent the majority of its time before departure in 2004 in the area north of Cape Mendocino after being tagged in the Gulf of the Farallones on 20 August 2004. It arrived at the Channel Islands again in late April 2005 but immediately turned south and spent May near Ensenada, Mexico. The whale returned to the Channel Islands in early June and spent most of its time there with occasional northward excursions to the Gulf of the Farallones, until mid-August when it again moved to the area north of Cape Mendocino, arriving in 2005 only one week earlier than in the previous year. The whale departed the EEZ on 12 November 2005, one week prior to its departure in 2004. A HR created from the period temporally overlapping in summer 2004 and 2005 was slightly larger in 2004 than 2005 (HR 20,800 km^2^ vs. 18,200 km^2^) but overall 66% of the 2005 HR overlapped with that in 2004 (Figure 6A). If we consider the largest contiguous HR areas from each year, 89% of the area from 2005 overlapped with that from the same period in 2004, showing very high site fidelity between years. The CAU in 2004 was almost twice the size of 2005 (4863 km^2^ vs. 2632 km^2^) and showed a lower percentage of overlap between years than the HRs with 47% of the 2005 CAU overlapping with the 2004 CAU (Figure 6B).

## Discussion

This data set represents the largest and most comprehensive collection of tracking data for any whale species. Considering that tagging occurred at several locales along the coast of California over multiple years, it is likely that this data set represents the majority of movement patterns of ENP blue whales off the U.S. West Coast. However, since ENP blue whales range widely [2], [5], [11], there may be other important areas along the coasts of Canada and Alaska, or further offshore, that may be less well represented in our data (Figures S1 & S2).

The blue whales in this study were generally concentrated in regions of typically high summer productivity along the upper continental slope from central to southern California. Summer upwelling throughout the California Current System produces high concentrations of euphausiids [44], while currents and bathymetric features aggregate them [45], [46], creating patches dense enough for blue whales to profitably feed on. The two areas of highest use were near the Gulf of the Farallones and at the western part of the Channel Islands. Both locations are sites of active upwelling, primary productivity, and krill [47], [48], [49], [50] and places where blue whales traditionally congregate [50]. Other regions of high krill density along central California presented in Santora et al. [47] also correspond to a large extent with the blue whale HRs and CAUs presented here, indicating a close association between high krill abundance and blue whale presence throughout the EEZ. The relatively low degree of overall CAU overlap compared to the amount of HR overlap throughout the tagged whales’ summer range suggests that, while blue whales return to the same broad area to forage in predictable prey habitat, they will focus their foraging effort in different fine-scale areas within this range based on where they find specific prey patches. A similar pattern was found for fin whales in the Mediterranean [51].

The northward progression of tagged whale locations later in the season (Figure 4) confirms the findings of Burtenshaw et al. [12] that the location of calling blue whales shifted northward from late summer into winter. The trend follows the timing of productivity generated by the spring bloom as it moves north along the California coast [12], [52]. Upwelling off southern California (33–36°N) occurs nearly year round, though at only moderate intensity [52]. The upwelling season occurs later and gets progressively shorter with increasing latitudes, and the greatest intensity of upwelling, and therefore, the region of highest productivity, occurs off northern California (36–42°N) [52]. Aggregations of euphausiids that are dense enough for blue whales to forage on have been shown to lag increases in primary productivity by 1–4 months [48], [53], [54]. The maximum rate of upwelling in the California Current System occurs from early-mid June off southern California to mid-August off northern California and Oregon [52], suggesting the whales may arrive in southern California early in the summer to forage on prey generated there by the moderate, early productivity until more intense upwelling to the north has developed and the density of prey increased. While the northern California portion of the California Current System produces greater upwelling intensity, the variability in timing and overall productivity of the region is much higher than the southern California portion [52], which may explain some of the observed variability between years in the timing and extent of the shift to more northern latitudes. Interestingly, while a few whales in this study traveled up to the waters off Washington, Vancouver Island, and Alaska, they all left well before late December, while Burtenshaw et al. [12] recorded blue whales in those areas until February or March in many years. This suggests significant inter-annual variability in use of the more northern latitudes, or that use of the area was not fully captured by the tracks presented here.

The lack of a statistical difference in HR and CAU areas between years was surprising given that foraging behavior occurred significantly farther north in some years [28] and line-transect surveys have observed blue whale distribution varying from year to year [55]. The California Current System is a highly variable ecosystem and blue whales might be expected to search over a larger area during years with lower productivity in order to find enough food [5]. This study captured the movements of blue whales during poor productivity years, including a severe El Niño event in 1998 [56], [57], [58] and another year of severely delayed and reduced upwelling in 2005 [59], and also during periods of very high productivity during La Niña events in 1999 and 2008 [60], [61]. It is possible that the relatively small and variable sample sizes across the years and high individual variability combined with the varying months of tag deployment obscured other underlying trends. Additional work correlating the whale tracks to environmental and prey data would be worthwhile to understand the factors driving the whales’ movements.

HR and CAU size varied by as much as almost two orders of magnitude among individuals within years, suggesting there were considerable behavioral differences among individual whales. In 2004, two HRs were approximately 36 times smaller than a third HR, which included both smaller HR areas. All three whales were tagged within four days of each other and had long duration tracks (76 and 156 d for the small HRs and 107 d for the large HR). Since feeding is the main factor driving blue whale distribution during the summer time [48], [50], the different HR areas appear to indicate that food requirements or ability to find prey may vary among individuals. While the method and scale that whales use to detect prey patches is unknown, they do vocalize while foraging [62], which can be heard up to 500 km away [63], so it is unlikely that ability to find prey was a factor. We therefore speculate that the difference in HR sizes represent different foraging strategies by individuals where some whales (represented by the smaller HRs) may choose to remain in a marginally productive (but known) prey patch while other whales (represented by the larger HRs) may choose to leave marginal prey patches in search of more productive areas. There is evidence suggesting that geographic foraging preferences also exist within the EEZ [2] and the different foraging strategies may reflect a preference for different prey species. While it is generally accepted that ENP blue whales in the California Current System will forage on both of the dominant euphausiid species *Thysanoessa spinifera* and *Euphausia pacifica* [48], [50], [64], there is some evidence for whales preferring one species [50] and the offshore waters used by some of the population (>30 miles [2]) are outside the typical range of *T. spinifera* [65]. A future project characterizing measures of productivity and feeding success could assess their influence on residence time.

While ENP blue whales are thought to forage throughout their migration range [28], they would be expected to maximize their food intake in the California Current System for as long as prey concentrations are favorable prior to departure. However, in this study it did not appear that the timing of EEZ departure varied with increased or decreased productivity on the foraging grounds. The only year when whales departed significantly later was 2004, when the North Pacific was affected by a weak El Niño, but the impacts were not noticeable in the California Current System [66]. There was no observable difference in departure date during the very strong 1998 El Niño event [56], [57], [58], and departure occurred slightly later in 2005, a year with severely delayed onset of upwelling [59]. Conversely, departure was not delayed during the extremely productive year of 1999 [60] when prey would have been plentiful. The measures of foraging effort used in this study (number of ARS patches per track, average ARS patch duration, longest ARS patch duration, duration of the last ARS patch prior to departure, and overall fraction of all track locations that were classified as ARS behavior) also did not significantly affect the departure date, suggesting that the whales foraged for as long as prey was available. Studies have shown that dense patches of euphausiids generally persist into October in the central and southern California Current System [48], [53] and likely later in the northern California Current System where the spring bloom starts later. The characteristics of individual prey patches, rather than overall prey density or availability, have been shown to predict the distribution of smaller marine predators [67], so blue whales may forage until the quality of available prey patches has been reduced below a threshold value.

The multi-year track of whale No. 3300840 provided an unprecedented opportunity to document year-to-year variability in migration timing and areas used by an individual blue whale. The record of this whale’s movements during the entire 2005 foraging season confirmed that the movements by the other whales presented here were representative of their behavior. The whale stayed in the southern portion of the California Current System during the late spring, where upwelling generally occurs earlier compared to further north [52]. Occasional excursions further north may have been investigations of the prey field in other areas. As summer progressed, the whale moved north, suggesting it was foraging on local increases in prey density generated by the northward progression of the spring bloom until it was time to migrate south again. The high degree of overlap between the 2004 and 2005 HRs showed that this whale had very strong site fidelity to particular areas despite year-to-year differences in the productivity of the California Current System. While the other whales in this study aggregated in places such as off the Channel Islands and the Gulf of the Farallones during multiple years, the precision of the timing of arrival to the area by whale No. 3300840 is remarkable and suggests that it was likely timed to coincide with a seasonal increase in abundance of zooplankton similar to what was observed by Visser et al. off the Azores Archipelago [54]. There was also remarkably similar timing of its departure from the EEZ in both years, considering the poorer productivity during 2005 [59] and the significantly later 2004 departures observed in other whales in this study.

**Figure 7:**
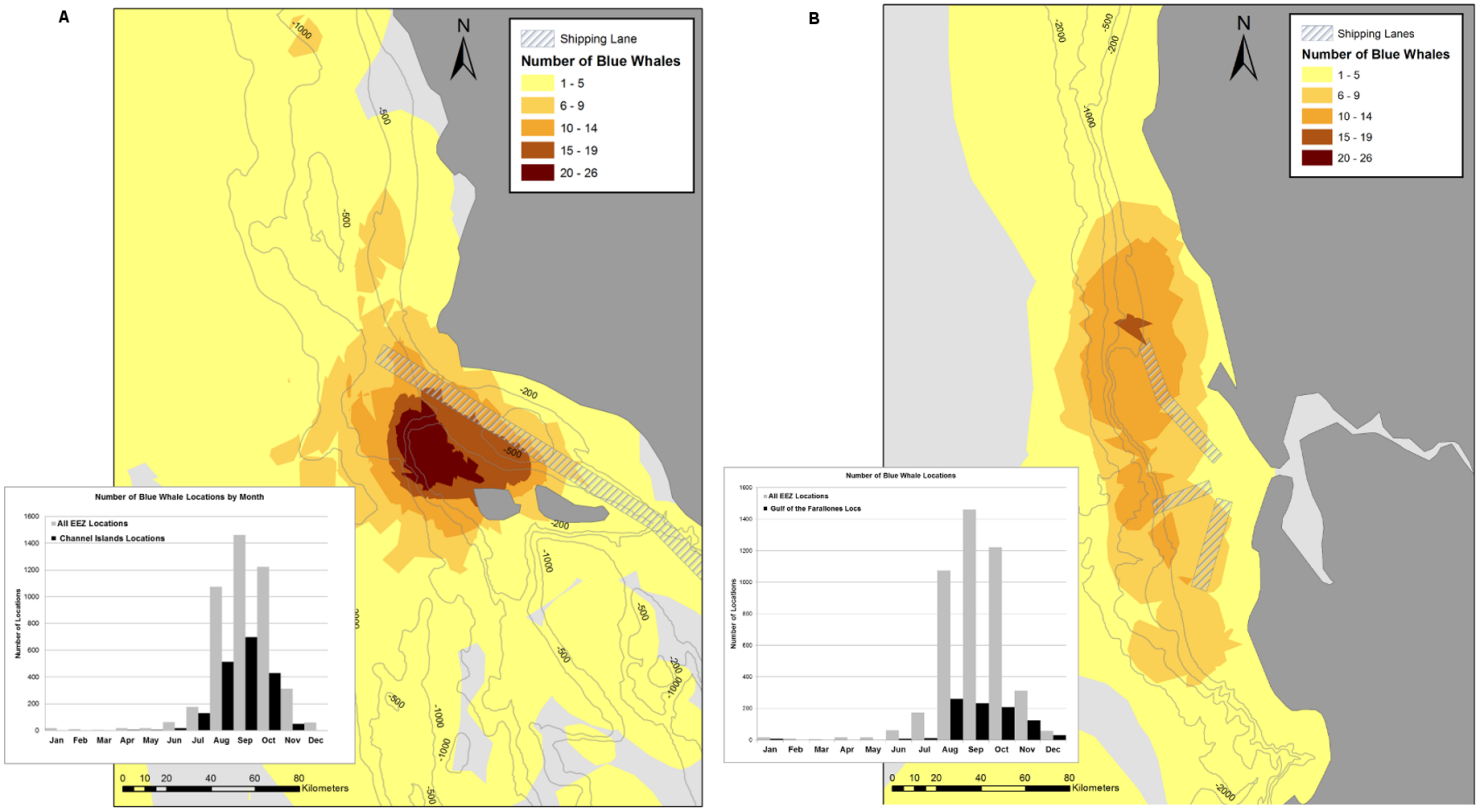
Number of overlapping blue whale Core Areas of Use near commercial shipping lanes. The Core Areas of Use were created from blue whale satellite tracks with > = 30 daily locations inside the U.S. Exclusive Economic Zone. The regions shown are the Channel Islands (A) and the Gulf of the Farallones (B). Tags were deployed off California from 1998–2008. Hashed polygons represent the commercial shipping lanes transiting the area. Inset histograms show the overall number of blue whale locations recorded in the U.S. Exclusive Economic Zone (gray), and the number of locations recorded in the area shown (black).

While the whales in this study generally occupied a wide region, most of the areas of highest concentration were close to large human population centers and busy port terminals. International shipping lanes transit through the two areas that were most heavily used from July to October (Channel Islands and the Gulf of the Farallones, Figure 7 A & B), raising the likelihood of anthropogenic impacts on the whales, either through increased underwater sound [8], [9], or through vessel collisions [10]. An assessment of ship-strike risk for three whale species, including blue whales, off southern California was recently conducted, concluding that blue whale ship-strike risk could not be reduced substantially in the area because the habitat-modeled densities were similar throughout southern California waters, and all shipping lane alternatives would cross moderate-to-high density areas [68]. This contrasts with the core areas identified from the telemetry data in our study. While we show that blue whales used the entire southern California waters, the high-use area at the western part of the Santa Barbara Channel suggests that moving the shipping route southwards would reduce the risk of ship strikes for blue whales, particularly during July to October.

Although blue whales in this study occurred all along the coast off central California, the greatest overlap among individuals was off Cordell Bank and along the 200–500-m isobaths. Closing the northern shipping lane heading to and from the ports in San Francisco Bay during August to November may help to reduce the likelihood of a ship strike in this area for blue whales. Another alternative would be to create one east-west lane that extends to at least the 2000-m isobath before branching so that it crosses the high-density areas as quickly as possible and in a limited area.

The comparatively higher spatial and temporal resolution of tracking data was well suited to identifying areas of high use relative to broader-scale ship-based surveys, especially in the case of predicting overlap with geographically small features like shipping lanes. These data also provide additional insight relevant to future habitat modeling effort, which may better explain some of the observed variability in home range size and migratory timing. The possible changes to shipping lanes identified here to minimize overlap with areas heavily used by tagged blue whales, demonstrate the utility of this type of data in providing relevant information to managers tasked with making necessary changes to reduce anthropogenic impacts on blue whales, while minimizing impacts to commercial and recreational interests. As they attempt to understand and mitigate whale-human interactions, the data presented here, in combination with existing survey results, will allow managers to understand where interactions are most likely to occur and how that likelihood changes seasonally and across years.

## Supporting Information

Figure S1 **Areas used by three blue whales that left and returned to the U.S. EEZ.** Kernel derived Home Ranges and Core Areas of Use were created from blue whale satellite tracking data within the U.S. Exclusive Economic Zone. Whales were tagged off California and traveled to Vancouver Island, British Columbia in 2004 (A), 2005 (B), and 2008 (C) before returning to the US Exclusive Economic Zone waters.
(TIF)

Figure S2 **Areas used by two blue whales that left and returned to the U.S. EEZ.** Kernel derived Home Ranges and Core Areas of Use were created from blue whale satellite tracking data within the U.S. Exclusive Economic Zone. Whales were tagged off California and traveled to the Gulf of Alaska in 2007 (A) and to the southern tip of Baja, Mexico in 2008 (B) before returning to the U.S. Exclusive Economic Zone waters.
(TIF)

Figure S3 **1998 individual overlapping 90% Home Range areas (A) and 50% Core Areas of Use (B).** Home ranges and Core Areas of Use were kernel derived from blue whale satellite tracks with > = 30 daily locations inside the U.S. Exclusive Economic Zone. Tags were deployed off California.
(TIFF)

Figure S4 **1999 individual overlapping 90% Home Range areas (A) and 50% Core Areas of Use (B).** Home ranges and Core Areas of Use were kernel derived from blue whale satellite tracks with > = 30 daily locations inside the U.S. Exclusive Economic Zone. Tags were deployed off California.
(TIFF)

Figure S5 **2004 individual overlapping 90% Home Range areas (A) and 50% Core Areas of Use (B).** Home ranges and Core Areas of Use were kernel derived from blue whale satellite tracks with > = 30 daily locations inside the U.S. Exclusive Economic Zone. Tags were deployed off California.
(TIFF)

Figure S6 **2005 individual overlapping 90% Home Range areas (A) and 50% Core Areas of Use (B).** Home ranges and Core Areas of Use were kernel derived from blue whale satellite tracks with > = 30 daily locations inside the U.S. Exclusive Economic Zone. Tags were deployed off California.
(TIFF)

Figure S7 **2006 individual overlapping 90% Home Range areas (A) and 50% Core Areas of Use (B).** Home ranges and Core Areas of Use were kernel derived from blue whale satellite tracks with > = 30 daily locations inside the U.S. Exclusive Economic Zone. Tags were deployed off California.
(TIFF)

Figure S8 **2007 individual overlapping 90% Home Range areas (A) and 50% Core Areas of Use (B).** Home ranges and Core Areas of Use were kernel derived from blue whale satellite tracks with > = 30 daily locations inside the U.S. Exclusive Economic Zone. Tags were deployed off California.
(TIFF)

Figure S9 **2008 individual overlapping 90% Home Range areas (A) and 50% Core Areas of Use (B).** Home ranges and Core Areas of Use were kernel derived from blue whale satellite tracks with > = 30 daily locations inside the U.S. Exclusive Economic Zone. Tags were deployed off California.
(TIFF)

Figure S10 **Latitude of blue whale locations from 1998 (A), 1999 (B), 2000 (C), and 2004 (D).** Locations used in the figure were from portions of blue whale satellite tracks that occurred within the U.S. Exclusive Economic Zone waters. The red line indicates the median value.
(TIF)

Figure S11 **Latitude of blue whale locations from 2005 (E), 2006 (F), 2007 (G), and 2008 (H).** Locations used in the figure were from portions of blue whale satellite tracks that occurred within the U.S. Exclusive Economic Zone waters. The red line indicates the median value.
(TIF)
