# Correction: Spatial and Temporal Occurrence of Blue Whales off the U.S. West Coast, with Implications for Management

**DOI:** 10.1371/journal.pone.0109485

**Published:** 2014-09-23

**Authors:** The PLOS ONE Staff

In the XML version of the published article, the legend for Figure 6 is incorrect. Figure 6 and its correct legend can be viewed here.

**Figure 6:**
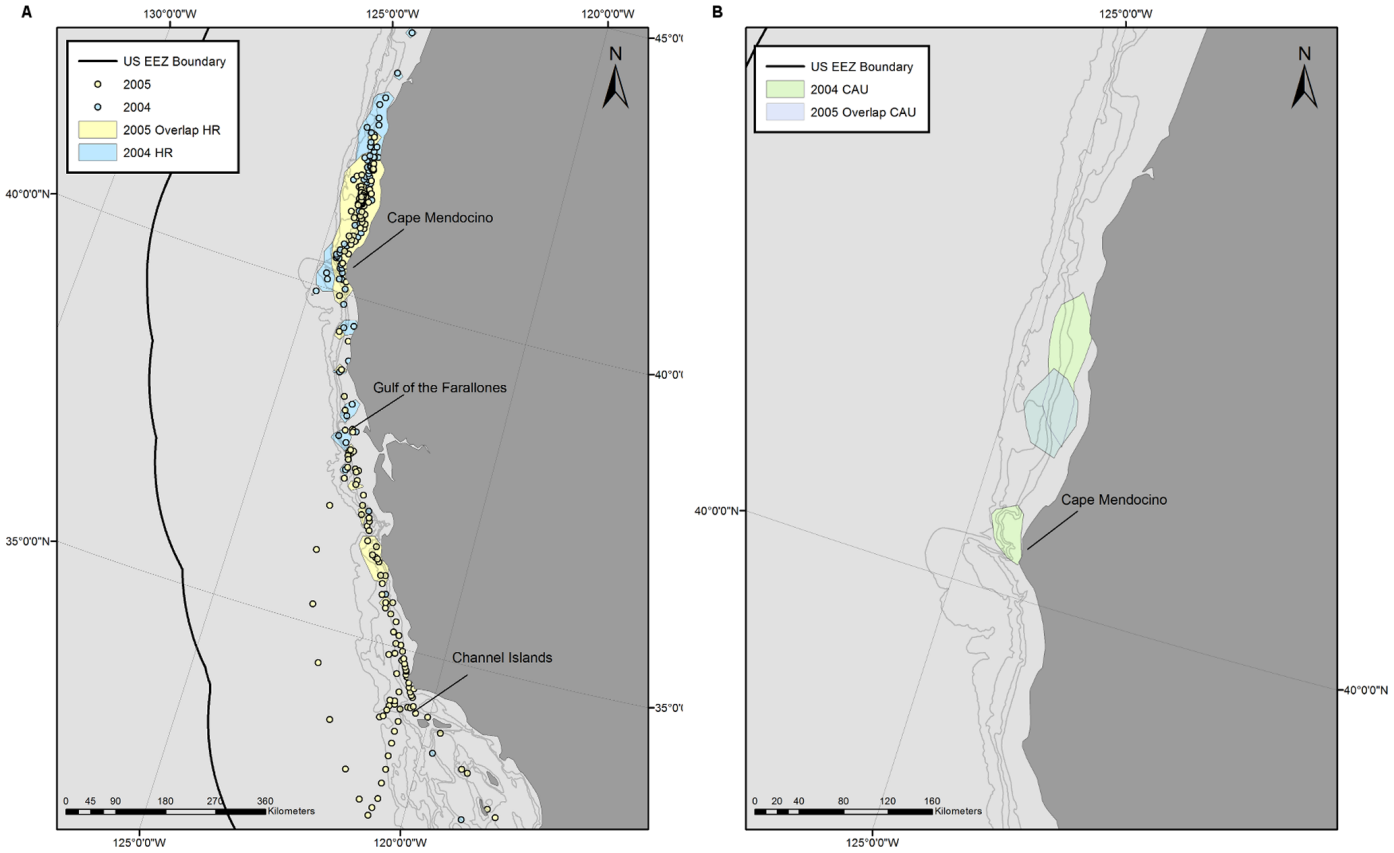
One whale was tracked for 504 d allowing for multi-year comparisons of space use. Locations within the U.S. Exclusive Economic Zone were recorded from 20 August to 12 November in both 2004 and 2005 for one whale. 90% Home Range (A) and 50% Core Areas of Use (B) were created from the locations to characterize the areas used by the whale in 2004 (blue) and 2005 (transparent yellow to better see overlap). The full summer tracks from 2004 and 2005 are included in the first image for reference.
