# Supplementary figures and images for: Spatial and Temporal Occurrence of Blue Whales off the U.S. West Coast, with Implications for Management
Source: PLoS One. 2014 Jul 23;9(7):e102959. doi: 10.1371/journal.pone.0102959 (PMC4108441; doi:10.1371/journal.pone.0102959)

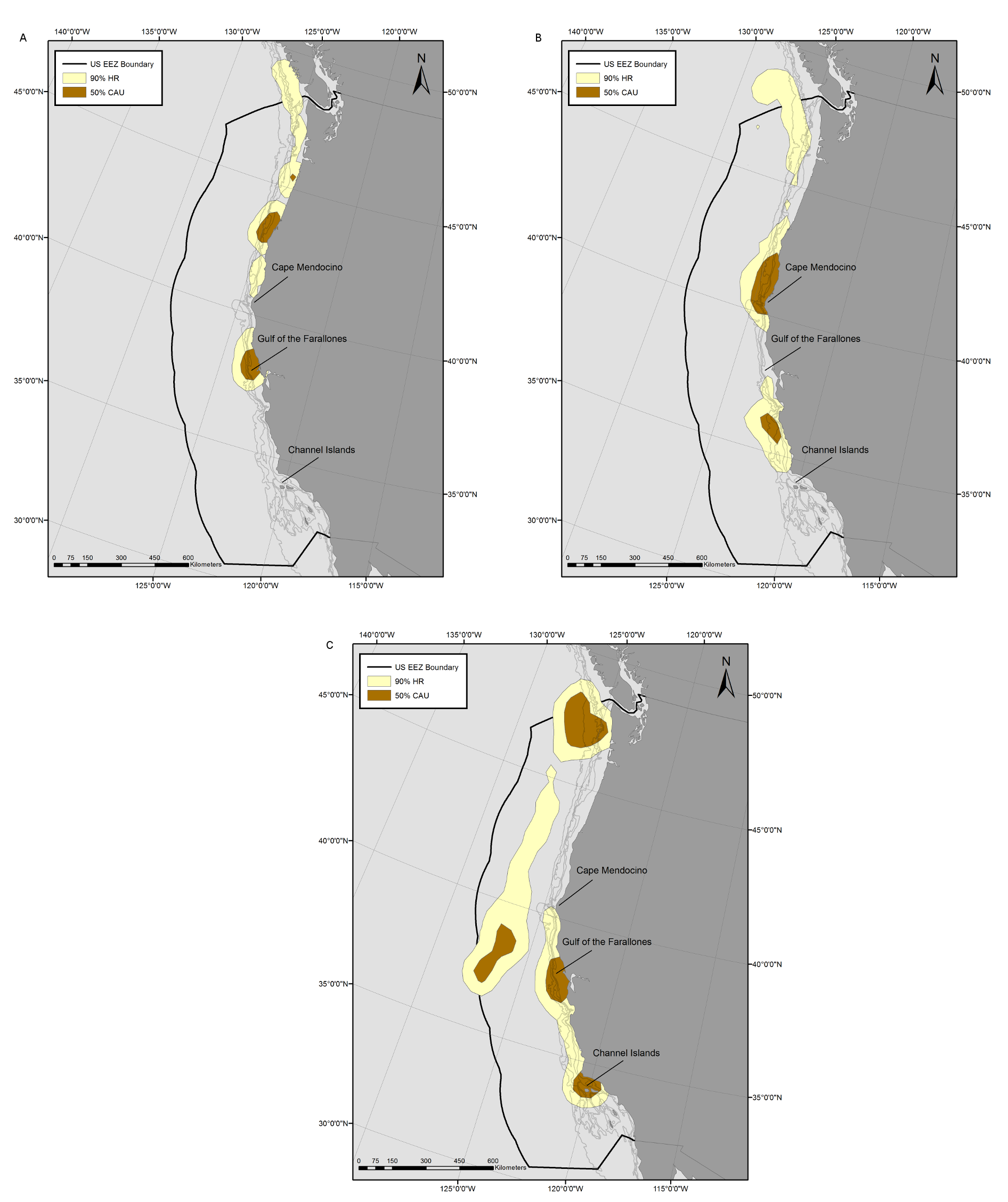

Supplement: Figure S1 — Areas used by three blue whales that left and returned to the U.S. EEZ. Kernel derived Home Ranges and Core Areas of Use were created from blue whale satellite tracking data within the U.S. Exclusive Economic Zone. Whales were tagged off California and traveled to Vancouver Island, British Columbia in 2004 (A), 2005 (B), and 2008 (C) before returning to the US Exclusive Economic Zone waters. (TIF) [file pone.0102959.s001.tif]

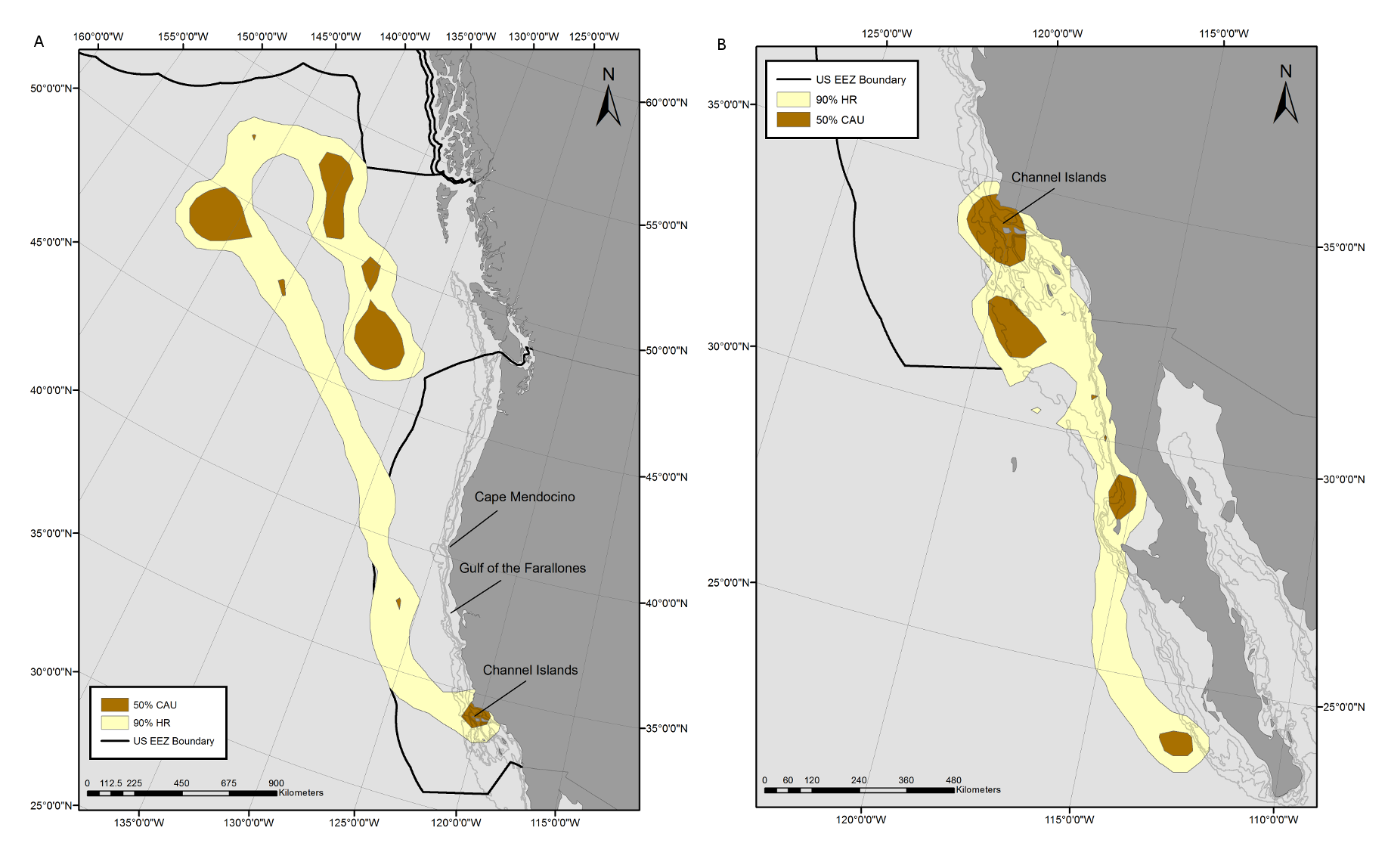

Supplement: Figure S2 — Areas used by two blue whales that left and returned to the U.S. EEZ. Kernel derived Home Ranges and Core Areas of Use were created from blue whale satellite tracking data within the U.S. Exclusive Economic Zone. Whales were tagged off California and traveled to the Gulf of Alaska in 2007 (A) and to the southern tip of Baja, Mexico in 2008 (B) before returning to the U.S. Exclusive Economic Zone waters. (TIF) [file pone.0102959.s002.tiff]

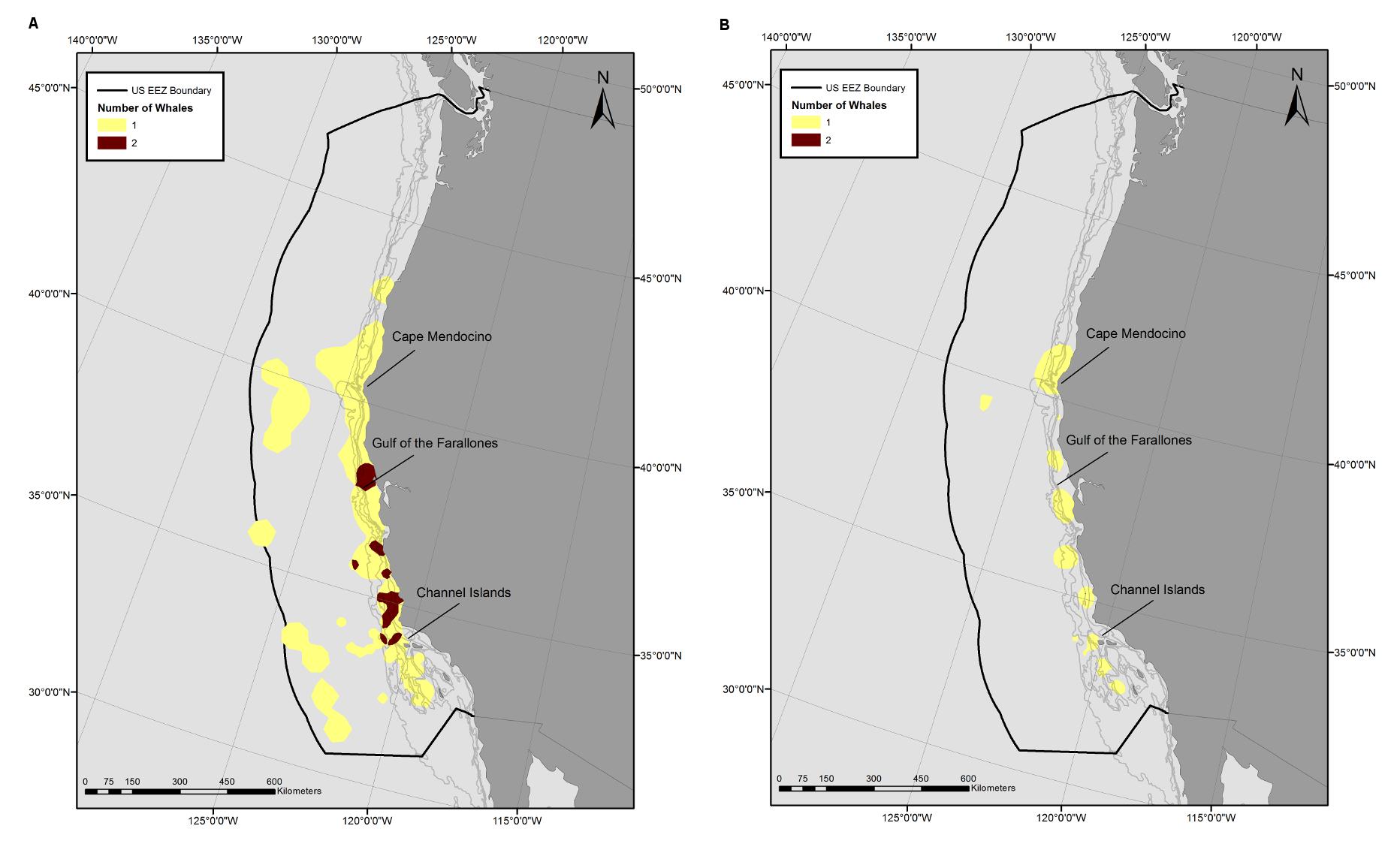

Supplement: Figure S3 — 1998 individual overlapping 90% Home Range areas (A) and 50% Core Areas of Use (B). Home ranges and Core Areas of Use were kernel derived from blue whale satellite tracks with > = 30 daily locations inside the U.S. Exclusive Economic Zone. Tags were deployed off California. (TIFF) [file pone.0102959.s003.tiff]

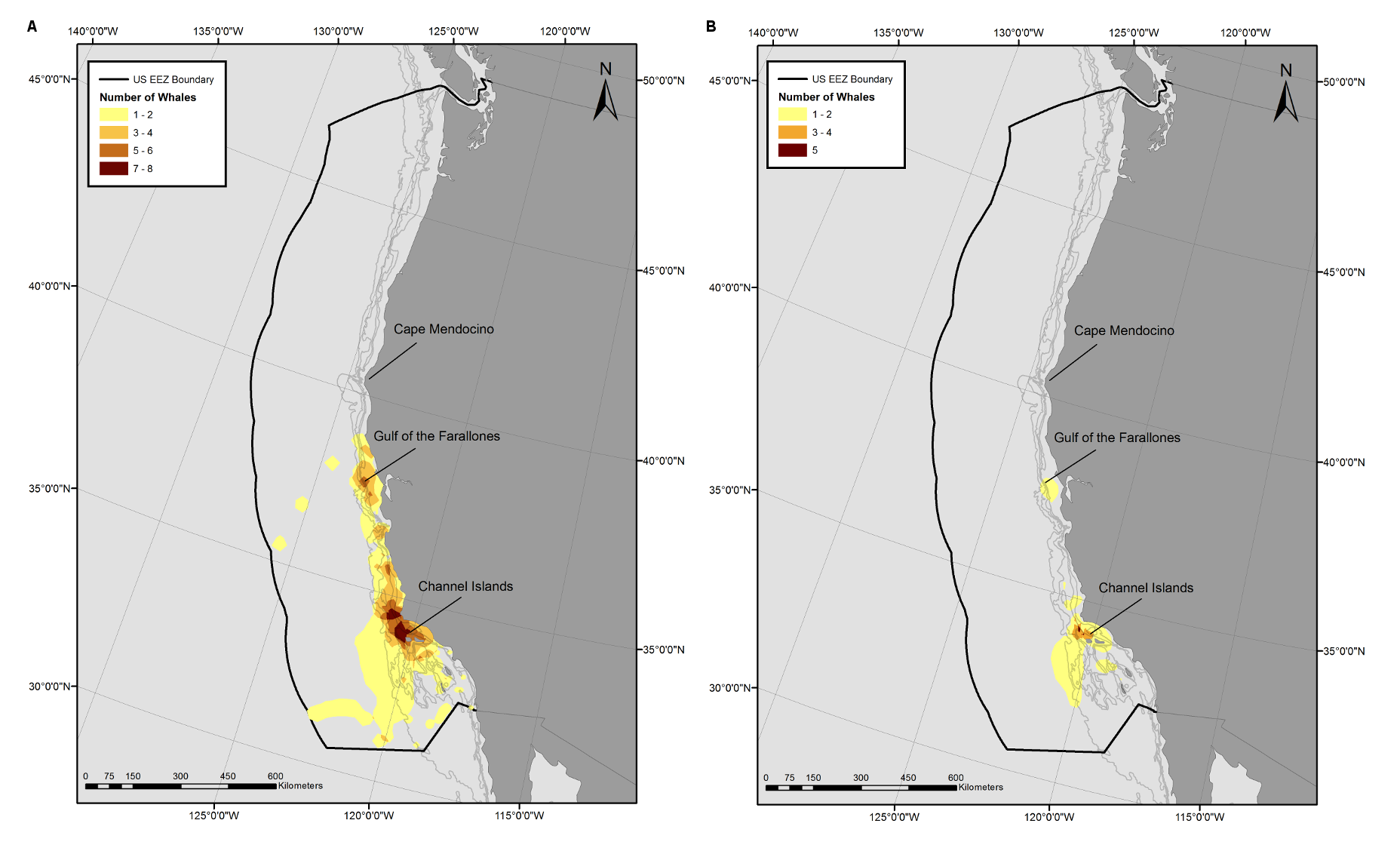

Supplement: Figure S4 — 1999 individual overlapping 90% Home Range areas (A) and 50% Core Areas of Use (B). Home ranges and Core Areas of Use were kernel derived from blue whale satellite tracks with > = 30 daily locations inside the U.S. Exclusive Economic Zone. Tags were deployed off California. (TIFF) [file pone.0102959.s004.tiff]

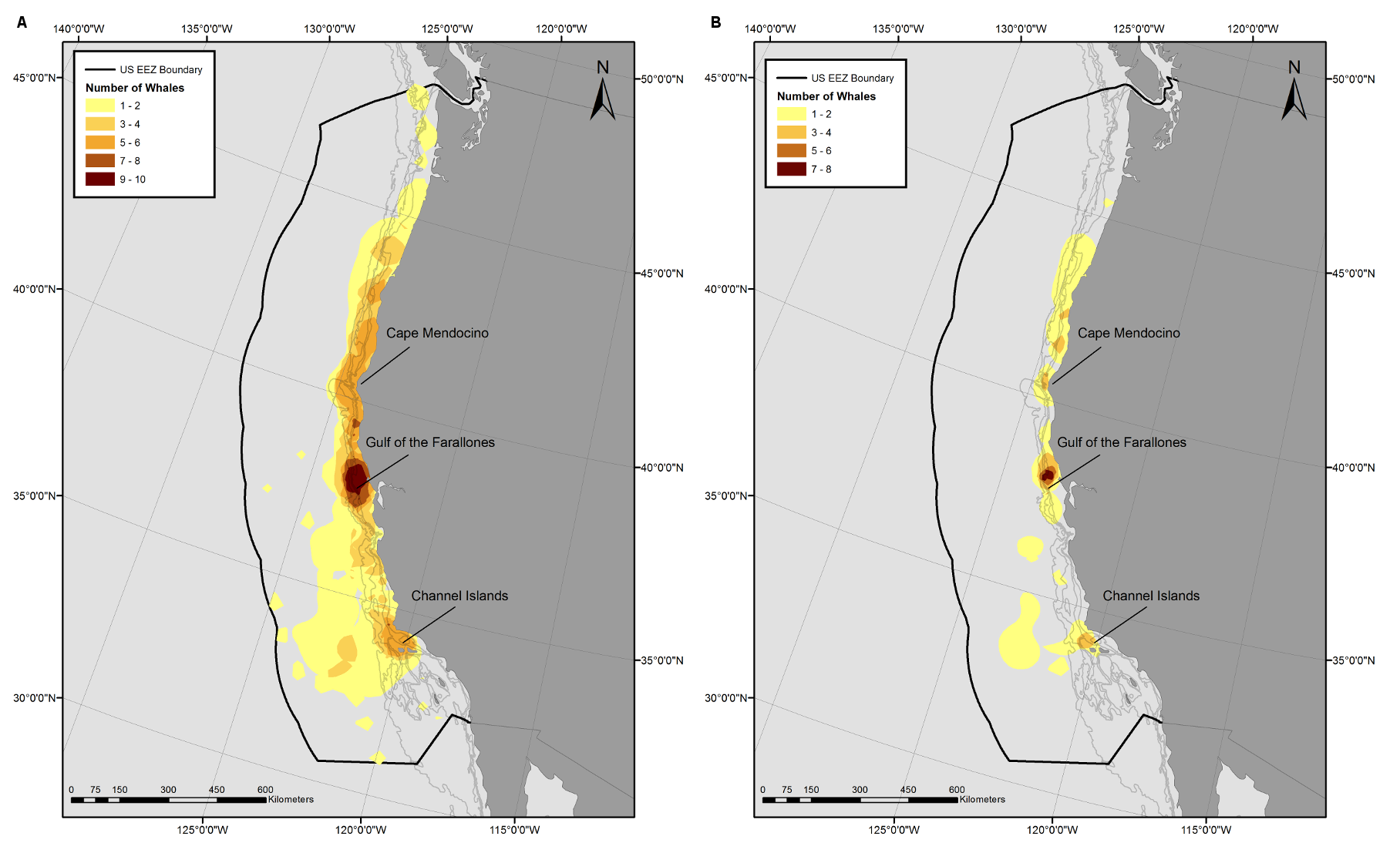

Supplement: Figure S5 — 2004 individual overlapping 90% Home Range areas (A) and 50% Core Areas of Use (B). Home ranges and Core Areas of Use were kernel derived from blue whale satellite tracks with > = 30 daily locations inside the U.S. Exclusive Economic Zone. Tags were deployed off California. (TIFF) [file pone.0102959.s005.tiff]

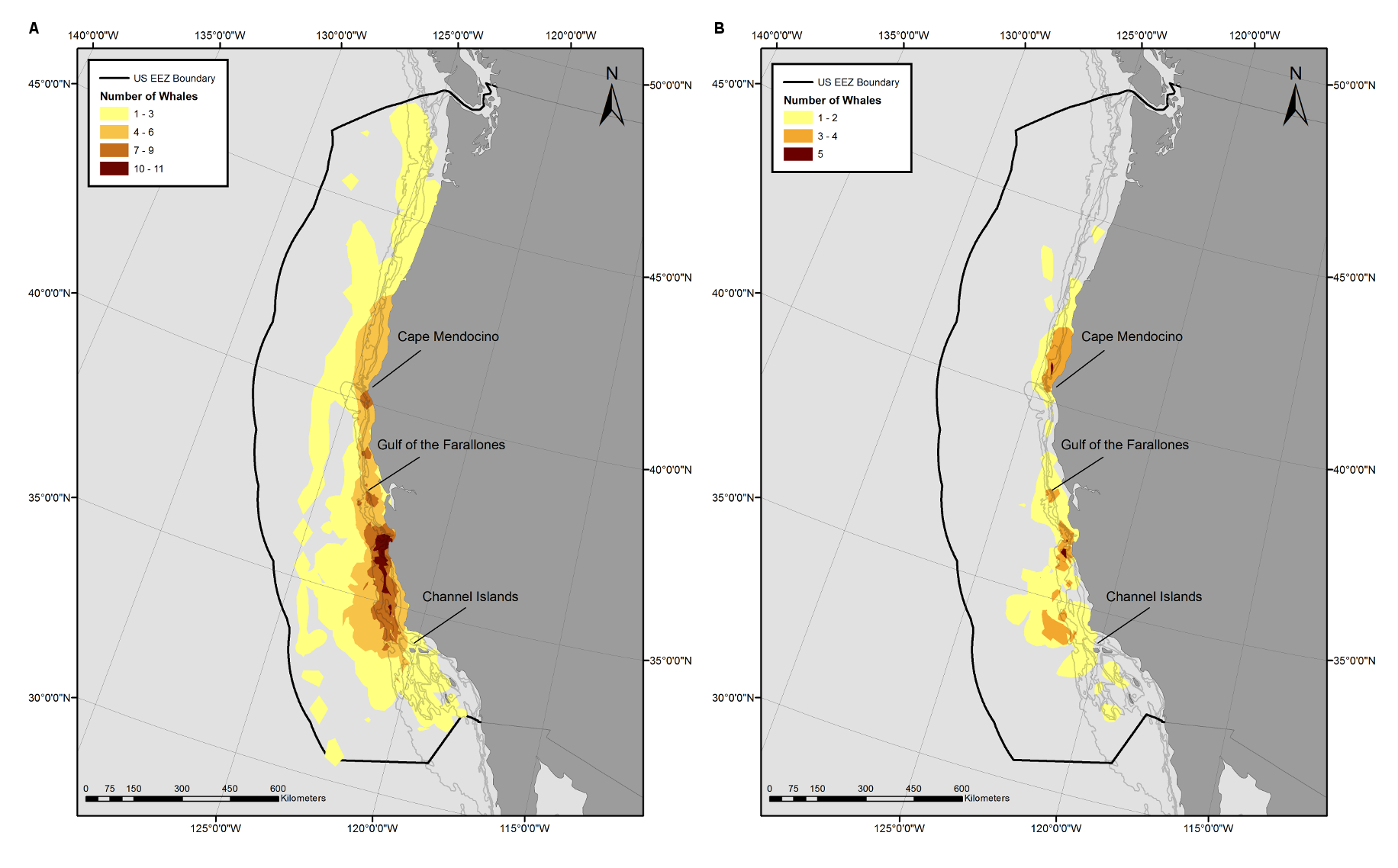

Supplement: Figure S6 — 2005 individual overlapping 90% Home Range areas (A) and 50% Core Areas of Use (B). Home ranges and Core Areas of Use were kernel derived from blue whale satellite tracks with > = 30 daily locations inside the U.S. Exclusive Economic Zone. Tags were deployed off California. (TIFF) [file pone.0102959.s006.tiff]

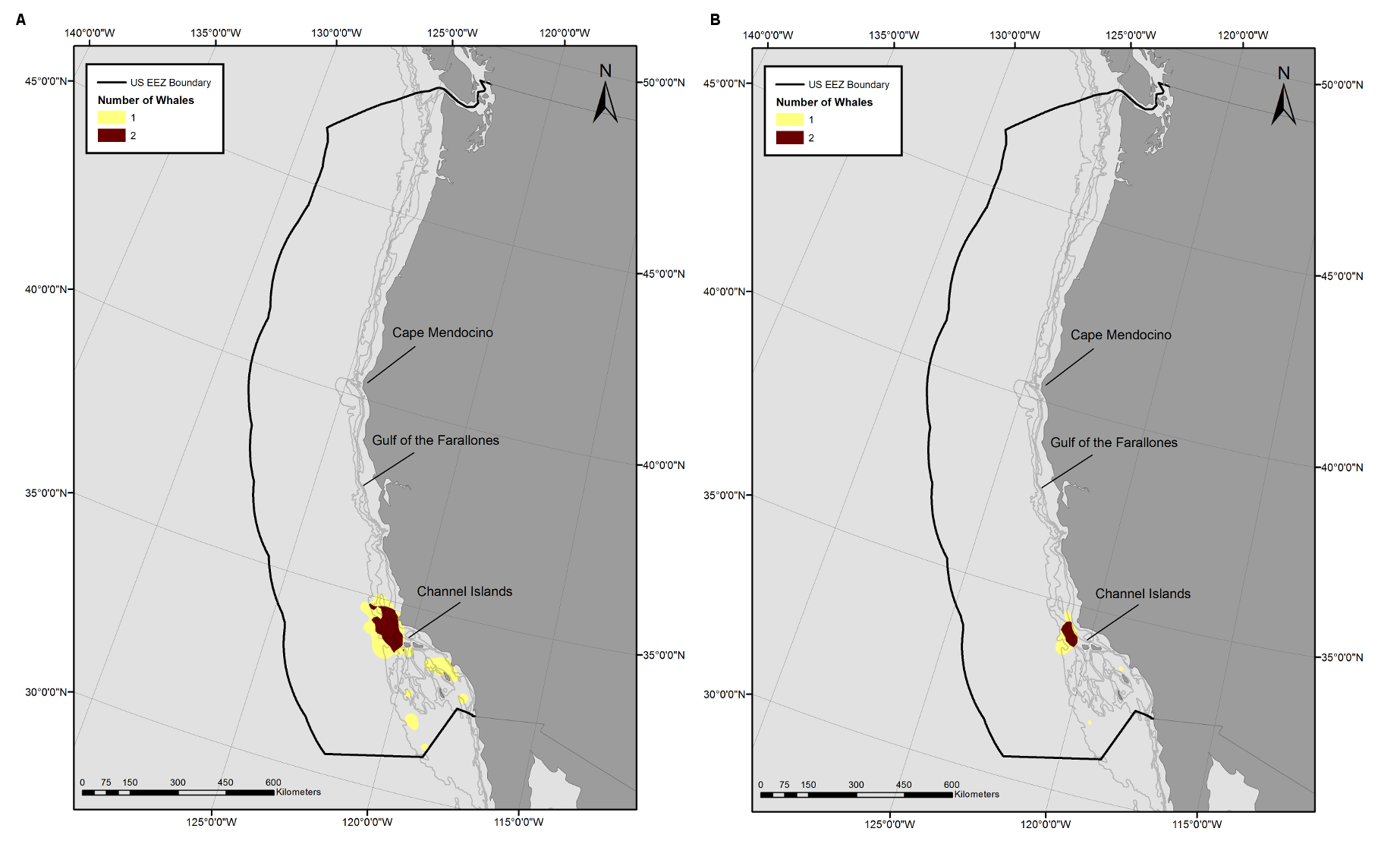

Supplement: Figure S7 — 2006 individual overlapping 90% Home Range areas (A) and 50% Core Areas of Use (B). Home ranges and Core Areas of Use were kernel derived from blue whale satellite tracks with > = 30 daily locations inside the U.S. Exclusive Economic Zone. Tags were deployed off California. (TIFF) [file pone.0102959.s007.tiff]

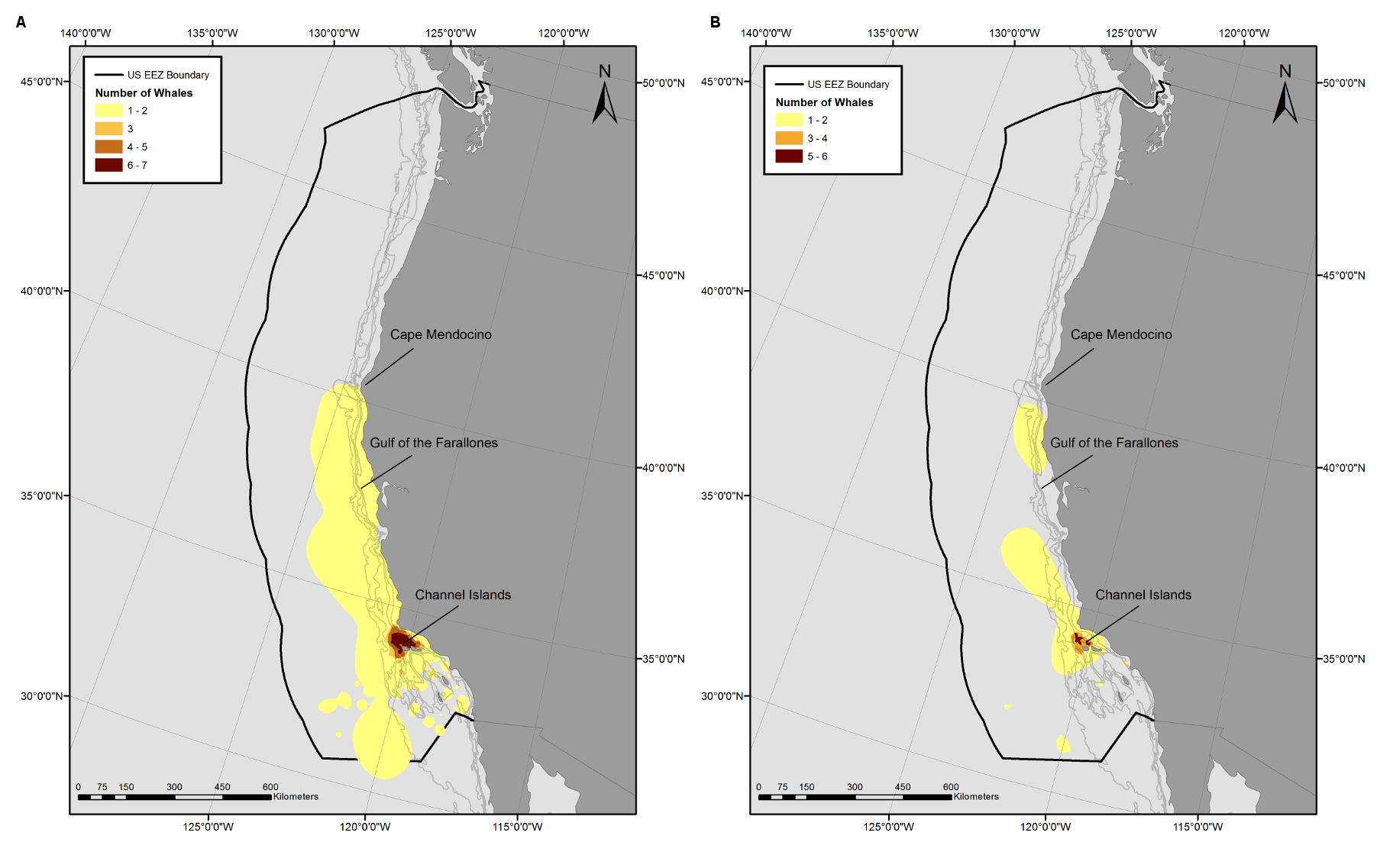

Supplement: Figure S8 — 2007 individual overlapping 90% Home Range areas (A) and 50% Core Areas of Use (B). Home ranges and Core Areas of Use were kernel derived from blue whale satellite tracks with > = 30 daily locations inside the U.S. Exclusive Economic Zone. Tags were deployed off California. (TIFF) [file pone.0102959.s008.tiff]

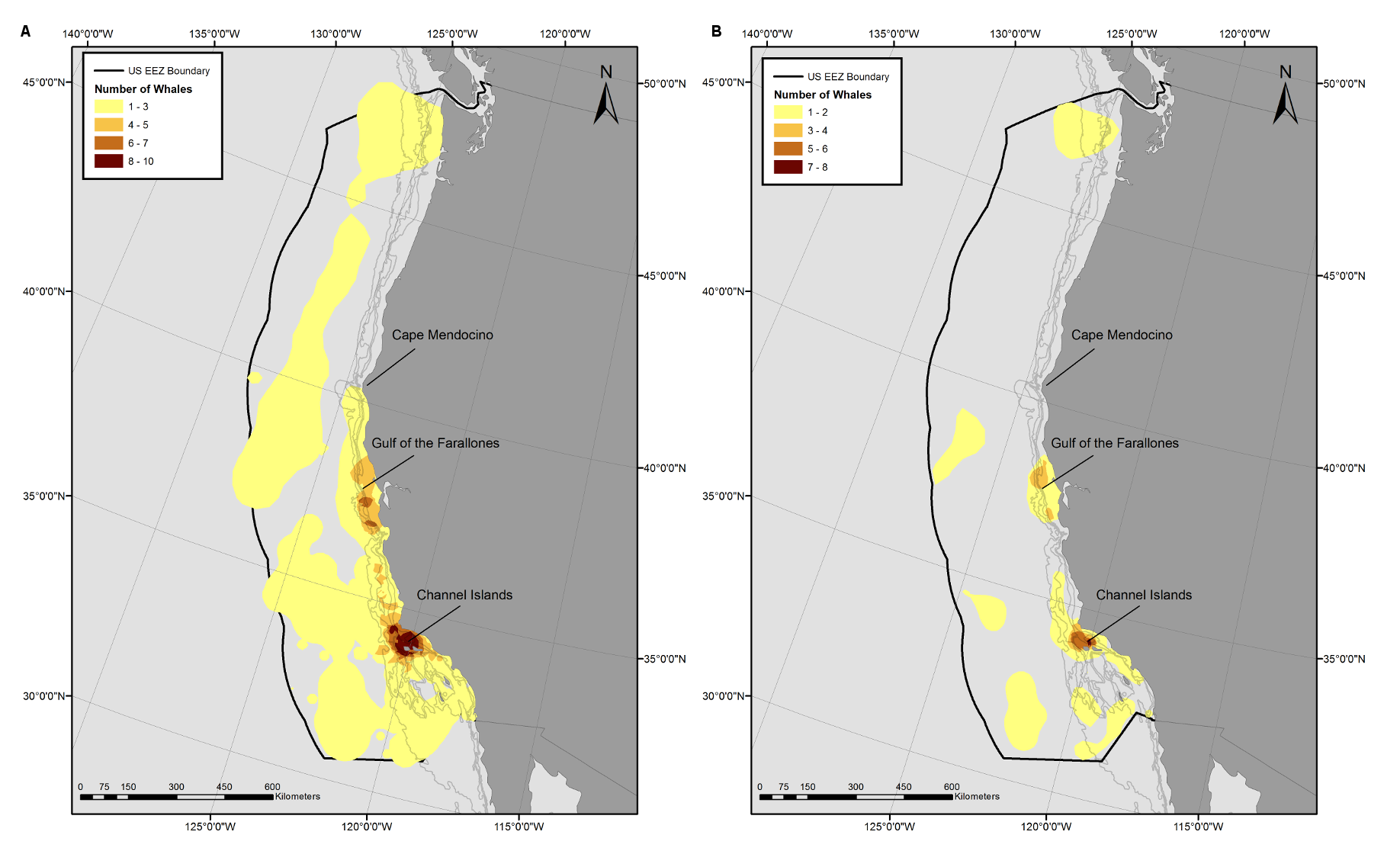

Supplement: Figure S9 — 2008 individual overlapping 90% Home Range areas (A) and 50% Core Areas of Use (B). Home ranges and Core Areas of Use were kernel derived from blue whale satellite tracks with > = 30 daily locations inside the U.S. Exclusive Economic Zone. Tags were deployed off California. (TIFF) [file pone.0102959.s009.tiff]

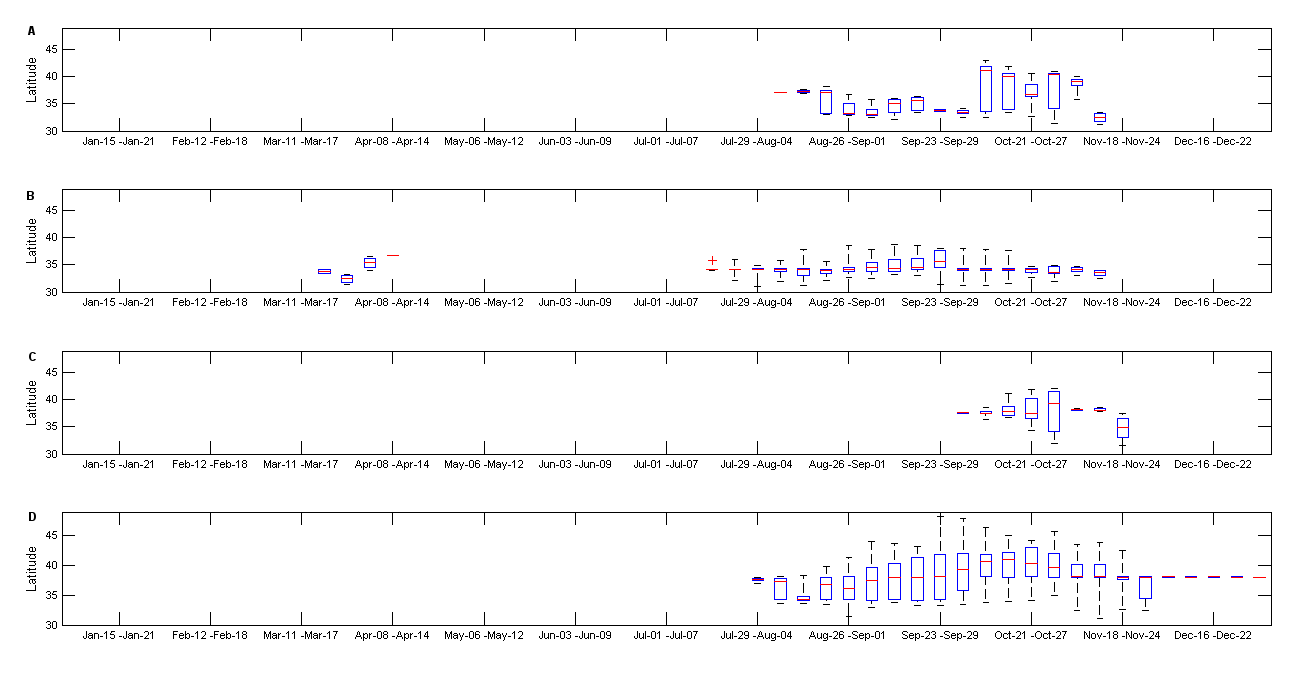

Supplement: Figure S10 — Latitude of blue whale locations from 1998 (A), 1999 (B), 2000 (C), and 2004 (D). Locations used in the figure were from portions of blue whale satellite tracks that occurred within the U.S. Exclusive Economic Zone waters. The red line indicates the median value. (TIF) [file pone.0102959.s010.tif]

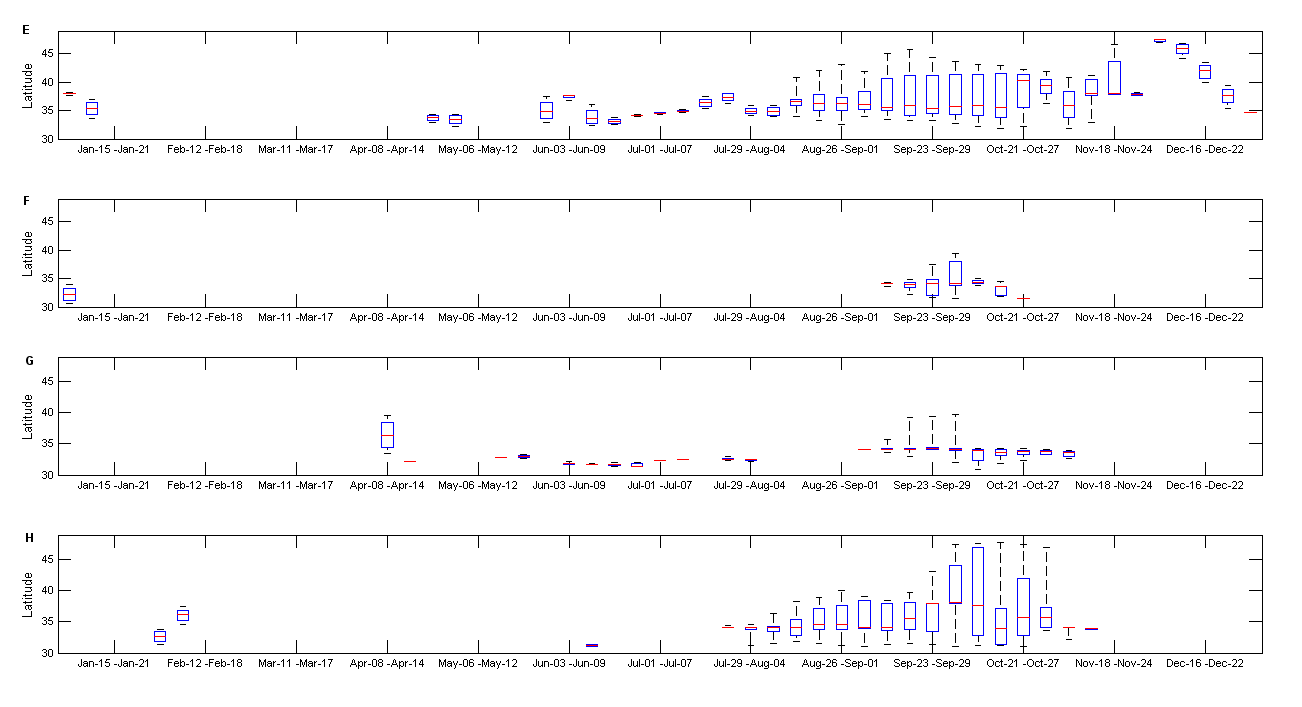

Supplement: Figure S11 — Latitude of blue whale locations from 2005 (E), 2006 (F), 2007 (G), and 2008 (H). Locations used in the figure were from portions of blue whale satellite tracks that occurred within the U.S. Exclusive Economic Zone waters. The red line indicates the median value. (TIF) [file pone.0102959.s011.tif]
